# Uterine Spindle Cell Sarcoma Presenting With Lower Gastrointestinal Bleeding: A Case Report

**DOI:** 10.7759/cureus.86486

**Published:** 2025-06-21

**Authors:** Ahmad Habbas, Ismail Althunibat, Shatha Elemian, Byron Okwesili, Abdul Aishat Abiola, Laith Sorour, Amro Al Radaideh, Andre Fedida

**Affiliations:** 1 Internal Medicine, Saint Michael's Medical Center, Newark, USA; 2 Gastroenterology and Hepatology, Saint Michael's Medical Center, Newark, USA; 3 Internal Medicine, Ascension Saint Francis Hospital, Evanston, USA; 4 Gastroenterology, Saint Michael's Medical Center, Newark, USA

**Keywords:** large pelvic mass, lower gi bleeding, sarcoma, spindle cell, uterine, weight loss

## Abstract

This case report presents a rare clinical manifestation of uterine spindle cell sarcoma, an aggressive and uncommon malignancy, in a 79-year-old female patient. The patient presented with lower gastrointestinal (GI) bleeding, constipation, and significant weight loss. Colonoscopy identified an infiltrative, ulcerated mass within the sigmoid colon, later confirmed by biopsy as a high-grade spindle cell sarcoma of uterine origin. Given the unresectable status of the mass, chemotherapy was initiated as the primary treatment. This report discusses the diagnostic challenges and highlights the importance of considering atypical presentations in patients with lower GI bleeding and weight loss.

## Introduction

Uterine sarcomas are uncommon malignancies, comprising less than 1% of all uterine cancers, with an estimated annual incidence of 0.36 to 0.64 cases per 100,000 women [1]. They are categorized into several histological subtypes, including leiomyosarcoma, endometrial stromal sarcoma (ESS), and undifferentiated uterine sarcoma (UUS) [2]. Spindle cell variants are particularly aggressive, with a tendency for early local invasion and metastasis.

The current World Health Organization classification recognizes four categories of endometrial stromal tumor: endometrial stromal nodule (ESN), low-grade endometrial stromal sarcoma (LG-ESS), high-grade endometrial stromal sarcoma (HG-ESS), and UUS [3]. Clinical manifestations often overlap with benign gynecological conditions, including abnormal uterine bleeding, pelvic pain, or rapidly enlarging uterine masses [4]. 

This case report documents an unusual presentation of uterine spindle cell sarcoma with lower gastrointestinal (GI) bleeding and discusses the challenges of diagnosis and management. 

## Case presentation

A 79-year-old woman with a past medical history of hypertension and paroxysmal atrial fibrillation presented to the gastroenterology clinic with a six-month history of intermittent lower GI bleeding, constipation, weight loss, and generalized fatigue. Her symptoms had worsened recently, prompting a referral for further evaluation. 

**Figure 1:**
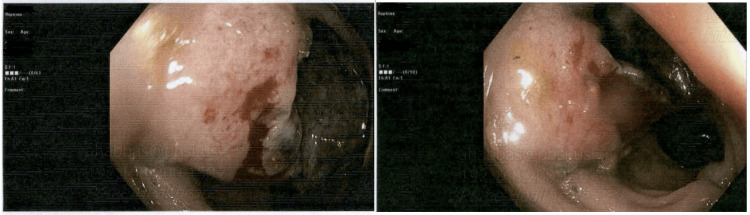
Colonoscopy images showing a fungating, infiltrative, and ulcerated mass in the sigmoid colon

Colonoscopy showed a fungating, infiltrative, and ulcerated mass in the sigmoid colon (Figure 1), approximately 26 cm from the anal verge. The lesion was partially circumferential, involving one-third of the luminal circumference without causing obstruction. Biopsies were obtained. 

Histopathology and immunohistochemistry

**Figure 2:**
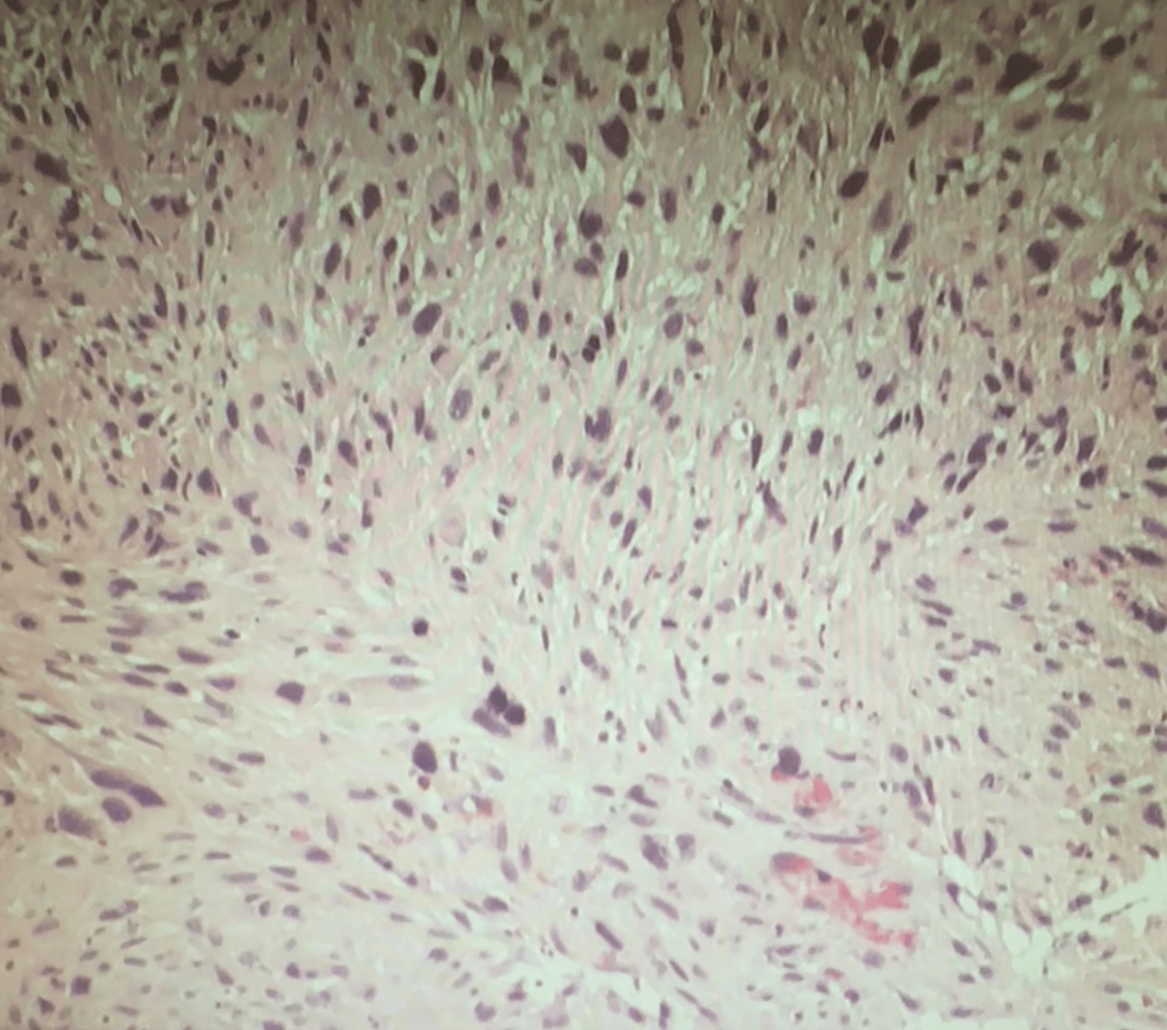
H&E-stained tissue section showing high-grade malignant tumor spindle cells infiltrating benign colonic mucosa H&E: Hematoxylin and eosin

**Figure 3:**
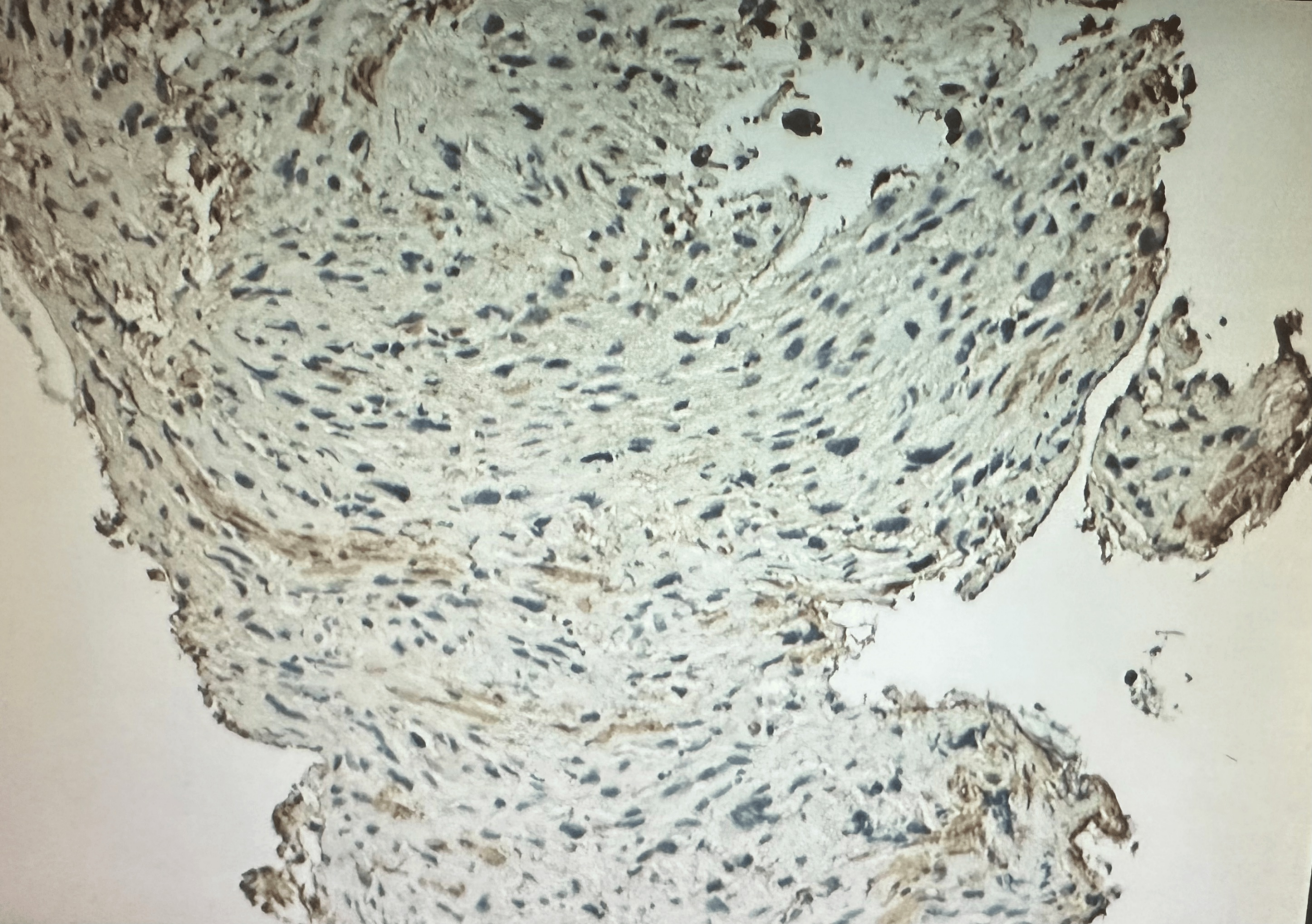
Positive focal SMA stain from the obtained tissue SMA: Smooth muscle actin

**Figure 4:**
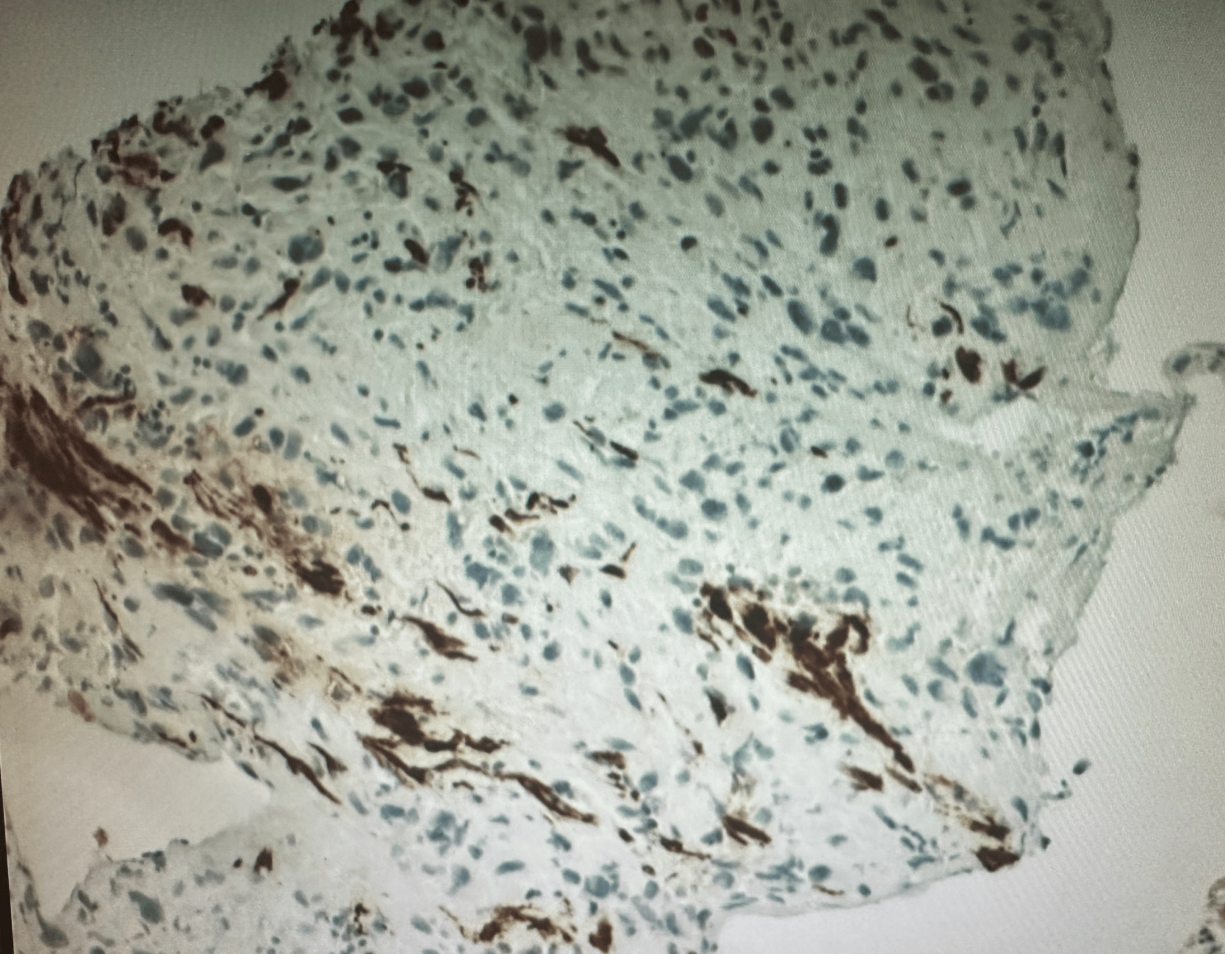
Positive focal desmin stain from the obtained tissue

Biopsy analysis showed a high-grade, poorly differentiated spindle cell malignant neoplasm with features suggestive of sarcoma. Tumor cells infiltrated benign colonic mucosa (Figure 2). Immunohistochemical staining showed positivity for GATA3, PAX8 (weak to moderate), beta-catenin (nuclear expression), MDM2 (some positive cells), and calretinin. The tumor displayed limited expression of smooth muscle actin (SMA) (Figure 3), desmin (Figure 4), WT1, synaptophysin, p40, and p16, with negativity for CK20, CDX2, and BAP1. This immunohistochemical profile supported the diagnosis of uterine sarcoma with spindle cell morphology. 

Imaging

**Figure 5:**
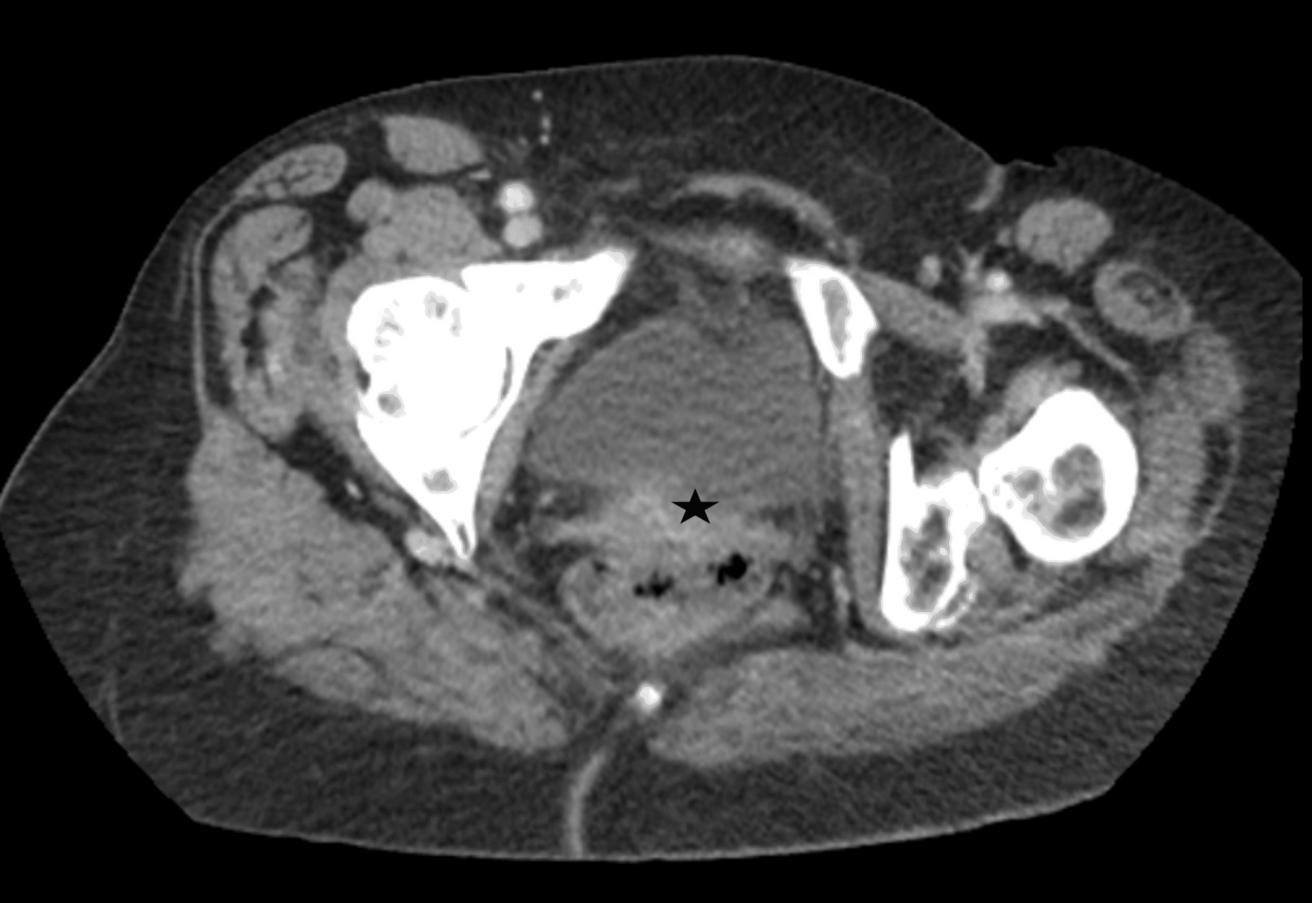
Enlarged pelvic mass invading the sigmoid colon with possible fistulous communication The star represents the uterine mass invading the rectum with possible fistula formation.

A contrast-enhanced CT scan of the abdomen and pelvis revealed a large pelvic mass, likely a conglomeration of the abnormal sigmoid colon and uterus, with possible fistulous communication (Figure 5). The mass compressed the left ureter, resulting in moderate hydronephrosis, and a 2.3 cm indeterminate nodule was noted on the right adrenal gland. No regional lymphadenopathy or definitive signs of distant metastasis were observed. 

Diagnosis

The diagnosis made was high-grade uterine spindle cell sarcoma with local invasion into the sigmoid colon. 

Therapeutic intervention

After multidisciplinary consultation, including gynecology and oncology, the tumor was deemed unresectable due to its local invasion and complex anatomy. Chemotherapy was initiated as the primary mode of management, aiming to control tumor progression, alleviate symptoms, and improve the patient’s quality of life. 

## Discussion

Uterine sarcomas are relatively rare, accounting for fewer than 5% of all uterine malignancies, yet they remain among the most aggressive gynecologic tumors due to their high risk of recurrence and poor overall survival rates [5]. Among these, spindle cell sarcomas represent a particularly uncommon subtype marked by rapid growth, early metastasis, and significant diagnostic challenges [6]. While patients classically present with gynecologic symptoms such as abnormal uterine bleeding or pelvic pain, atypical manifestations - including lower GI bleeding - can occur if the tumor invades or extends into surrounding structures, such as the sigmoid colon in this case. 

Histopathological and immunohistochemical evaluations are crucial for distinguishing uterine spindle cell sarcomas from other spindle cell neoplasms in the GI tract, as the morphological overlap can be substantial [5,7]. Markers indicative of Müllerian origin - such as PAX8 and GATA3 - are key to confirming a uterine primary, particularly when the lesion appears in an unusual location. In this patient, the tumor’s extension into the sigmoid colon and the presenting symptom of lower GI bleeding highlight the potential for uterine sarcomas to manifest similarly to primary colorectal tumors, complicating and potentially delaying the diagnostic process. 

Imaging modalities, including CT and MRI, play a vital role in defining the anatomic extent of the disease and evaluating resectability [7]. In advanced cases with significant local invasion, such as the one described, primary surgical management may not be feasible. Instead, chemotherapy becomes the mainstay of treatment, aiming to control tumor progression and provide palliation [8]. However, the prognosis for high-grade uterine sarcomas remains guarded, and the response to conventional chemotherapy is variable. 

As this case demonstrates, an older patient presenting with GI symptoms, especially unexplained bleeding and weight loss, warrants a broad differential diagnosis that includes gynecological malignancies, even when the initial findings point to a GI source. Prompt recognition, multidisciplinary evaluation, and the integration of pathology, imaging, and immunohistochemistry are essential to ensuring accurate diagnosis and effective management. Future investigations into novel therapeutic targets and personalized treatment regimens are critical, given the limited efficacy of standard therapies in advanced uterine sarcomas [6,8].

## Conclusions

This case emphasizes the diagnostic challenges associated with high-grade uterine spindle cell sarcomas presenting with non-gynecologic symptoms such as lower GI bleeding. The patient’s course highlights the need for a high index of suspicion and comprehensive workup in older patients with atypical GI presentations. Continued research is essential to improve therapeutic outcomes in this aggressive malignancy. 
